# Effect of bite force in occlusal adjustment of dental implants on the distribution of occlusal pressure: comparison among three bite forces in occlusal adjustment

**DOI:** 10.1186/s40729-015-0014-2

**Published:** 2015-06-03

**Authors:** Sho Kayumi, Yoshiyuki Takayama, Atsuro Yokoyama, Nana Ueda

**Affiliations:** 1 Removable Prosthodontics, Hokkaido University Hospital, Hokkaido University, Kita-14, Nishi-5, Kita-Ku, Sapporo, 060-8648 Japan; 2 Department of Oral Functional Prosthodontics, Division of Oral Functional Science, Graduate School of Dental Medicine, Hokkaido University, Kita-13, Nishi-7, Kita-Ku, Sapporo, 060-8648 Japan

**Keywords:** Implants, Occlusal adjustment, Nonlinear finite element analysis

## Abstract

**Background:**

The purpose of this study was to investigate the influence of occlusal forces (the contractile force of masticatory muscles) exerted during occlusal adjustment on the distribution of the forces among teeth, implants, and temporomandibular joints (TMJs) in intercuspal clenching in cases with bilateral missing molars and premolars by using finite element analysis.

**Methods:**

A three-dimensional finite element model of the mandible with eight implants in the premolar and molar regions was constructed. Linearly elastic material properties were defined for all elements except the periodontal ligament, which was defined as nonlinearly elastic. The TMJs and antagonists were simplified and replaced with nonlinear springs. Antagonists were assumed to be natural teeth or implants and had two- or three-stage displaceability. We constructed finite element (FE) models in which occlusal adjustment with three kinds of occlusal force (40 N as a light bite, 200 N as a hard bite, and 400 N as a maximum biting force) was performed. The clearance by occlusal adjustment was decided beforehand with a trial-and-error method so that the occlusal forces were distributed similarly to the distribution of the natural dentition. Each model was evaluated under loads of 40, 100, 200, 400, and 800 N to determine the distribution of occlusal forces on the teeth and implants.

**Results:**

The occlusal forces were concentrated on the most posterior implants while the load was larger, and the percentage of bearing force at the TMJ was small, and vice versa.

**Conclusions:**

Maximum biting force was better for occlusal adjustment to prevent overloading of the most posterior implant.

## Background

Dental implants have been widely used to restore or maintain occlusion, function, and esthetics and are particularly effective for partially edentulous jaws [1]. However, the difference of the displaceability of the implants and natural teeth with periodontal ligaments (PDLs) [2] may cause a problem in an arch that includes both implants and teeth. There is controversy about whether this difference should be considered in occlusal adjustment. Misch [3] stated that a clearance equivalent to the displaceability of the PDL should be allowed for the occlusal surfaces of implant-retained prostheses to prevent stress concentration. Contrastingly, Miyata et al. [4] stated that occlusal contact in implants should be equal to that of natural teeth to maintain the stomatognathic system. Kasai et al. [5] reported that hard biting appeared to be better for occlusal adjustment to avoid overloading of the most posterior implant in unilateral distal extension. However, when the occlusal load is mainly supported by implants, it has not been clarified whether the occlusal adjustment of the implants should be done as in the case of natural dentition. Moreover, in such cases, it is also necessary to consider the far lower displaceability of implants than that of temporomandibular joints (TMJs) in the stomatognathic system.

The purpose of this study was, therefore, to investigate the influence of occlusal forces (the contractile force of masticatory muscles) exerted during occlusal adjustment on the distribution of forces among teeth, implants, and TMJs during intercuspal clenching in cases with bilateral missing molars and premolars by using finite element analysis.

## Methods

### Finite element model

**Fig. 1:**
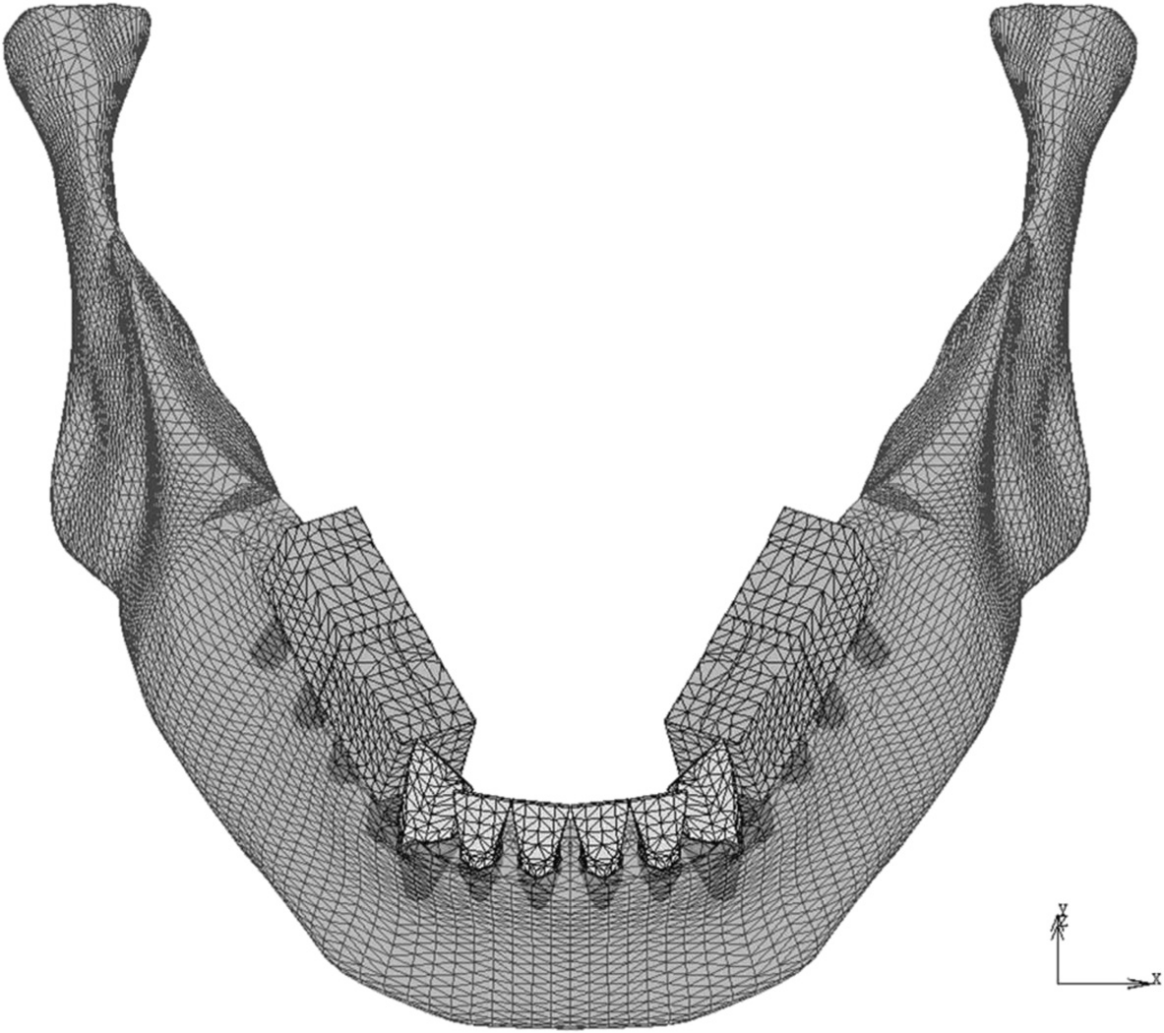
Finite element models (model-I and model-T). The tooth roots and the implant bodies are displayed with permeability

Three-dimensional finite element (FE) models were based on those reported by Kasai et al. [5] and consisted of a mandible, natural teeth with periodontal ligaments, and titanium implants with superstructures. All elements were homogenous and isotropic. In the models, eight implants replaced all of the premolars and molars (Fig. 1).

The mass/volume and the shape of the mandible were assumed to be 2 and B, respectively, according to the classification of Lekholm and Zarb [6]. The implant fixtures were 3.75 mm in diameter and 10 mm in length [7]. The dimensions of the natural teeth and periodontal ligaments were based on the literature [8–10]. The surface area of the periodontal ligament (PDL) corresponded to the anatomical value [10], and its thickness was 0.25 mm at all sites. The occlusal surfaces of the implants and the teeth were simplified and flattened in agreement with Monson’s sphere. The FE model consisted of approximately 42,000 nodes and 210,000 tetrahedral elements.

**Fig. 2:**
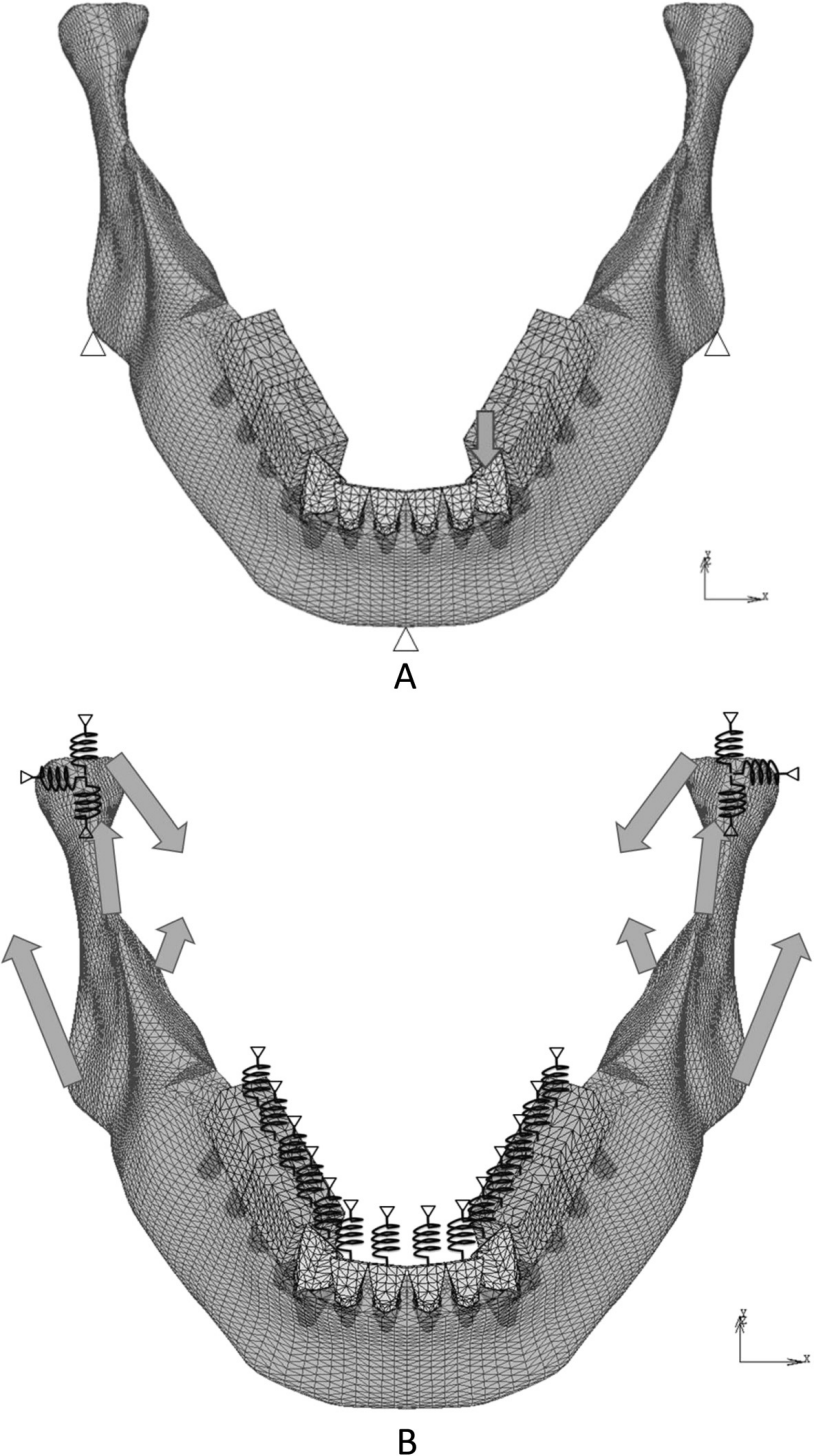
Boundary conditions to verify the displaceability of teeth (**a**) and analyze the distribution of occlusal forces (**b**). *Arrows*: loads, *triangles*: restricted nodes, *zigzags*: springs

The properties of the materials, except for the PDL, were based on previous studies [11–15] (Table 1). The biphasic properties for the PDL were determined according to the literature [2, 3, 16, 17]. The PDL was assigned two-phase properties. Young’s modulus and Poisson’s ratio were 0.33 MPa and 0.3 for phase 1, respectively. For phase 2, they were 16 MPa and 0.45, respectively. Phase 2 was applied when the von Mises stress exceeded 0.025 MPa. The load-displacement curve of the teeth was verified with the analysis described below (Fig. 2a).

**Table 1:** Material properties

Materials	Modulus of elasticity (MPa)	Poisson ratio
Enamel	80,000	0.3
Dentin	17,600	0.25
Inplant (titanium)	117,000	0.32
Superstructure (gold alloy)	94,000	0.3
Cortical bone	14,000	0.3
Cancellous bone	7,900	0.3

### Boundary conditions of the model and simulation of occlusal adjustment

The boundary conditions used to verify the displaceability of teeth and analyze the distribution of occlusal forces are shown in Fig. 2a, b, respectively. In the former model, a vertical load was applied to the left canine with the restriction of nodes on the bottom of the mandible (Fig. 2a). FE analysis was performed under various loads following the construction of a load-displacement curve. In the FE models used to analyze the distribution of occlusal force, TMJs, maxillary teeth, and maxillary implants were replaced with appropriate springs to simplify the model (Fig. 2b).

The antagonists of the mandibular anterior teeth were assumed to be natural teeth, and those of mandibular implants were assumed to be either teeth or implants. According to the condition of the antagonists of the mandibular implants, the models with opposing natural teeth and implants were designated model-T and model-I, respectively.

**Fig. 3:**
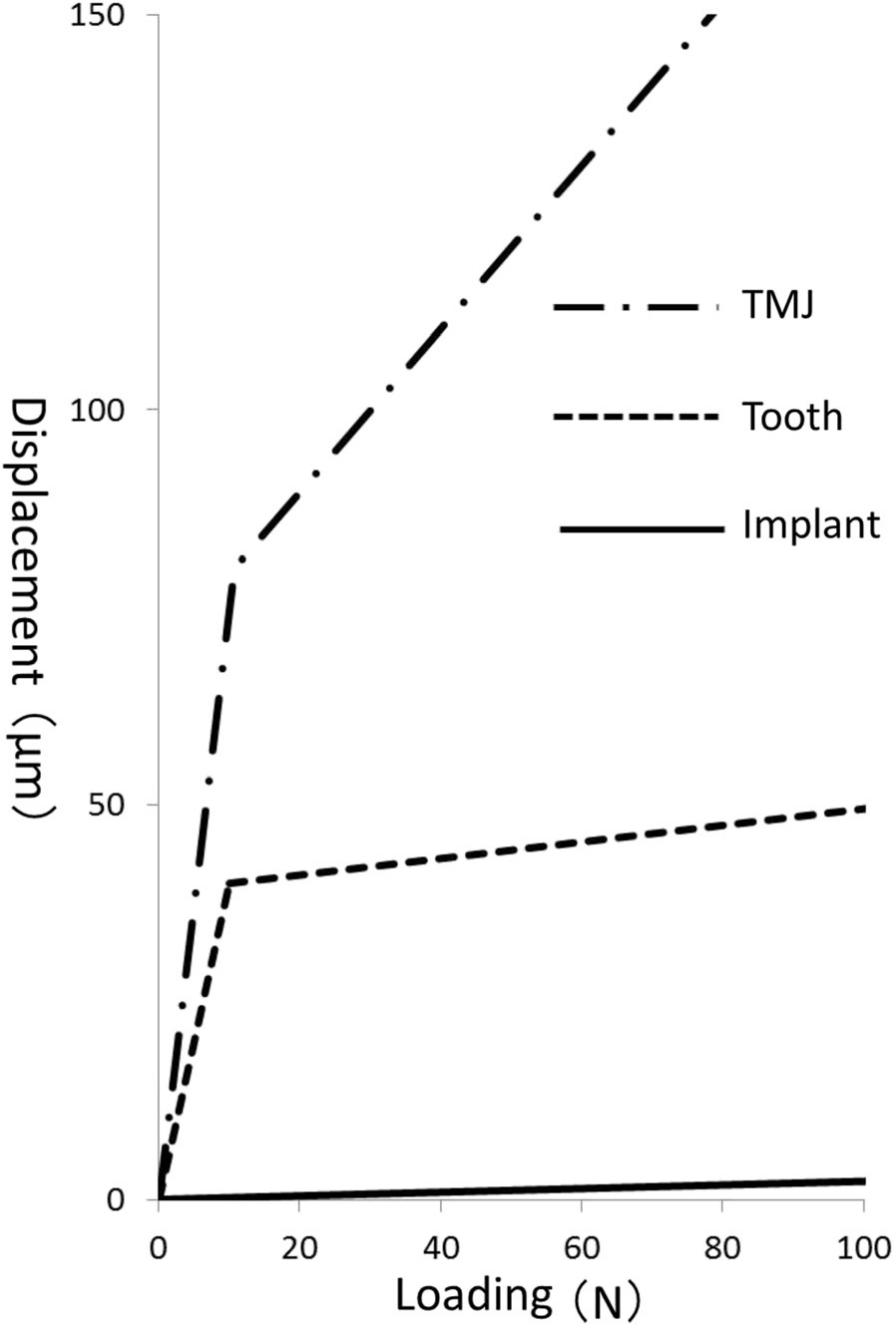
Load-displacement curves of the springs

The springs for the maxillary teeth or implants, except for the anterior teeth, were directed perpendicular to the occlusal plane. Each of those springs linked an external restricted node to the node corresponding to the occlusal central pit on a mandibular tooth, which allowed displacement perpendicular to the occlusal plane. The springs for temporomandibular joints linked an external restricted node to the top of the mandibular condyle. Nonlinear characteristics according to the load-displacement curves of the teeth [2, 3, 16, 17] and cartilage [18] were given to the springs of the opposing teeth and TMJs, respectively. The springs for maxillary implants had linear compression characteristics. The springs for antagonists had little resistance under tension to simulate detachment. The properties of these springs were confirmed by load-displacement curves (Fig. 3) obtained using a simple FE model consisting of an element and a spring.

**Fig. 4:**
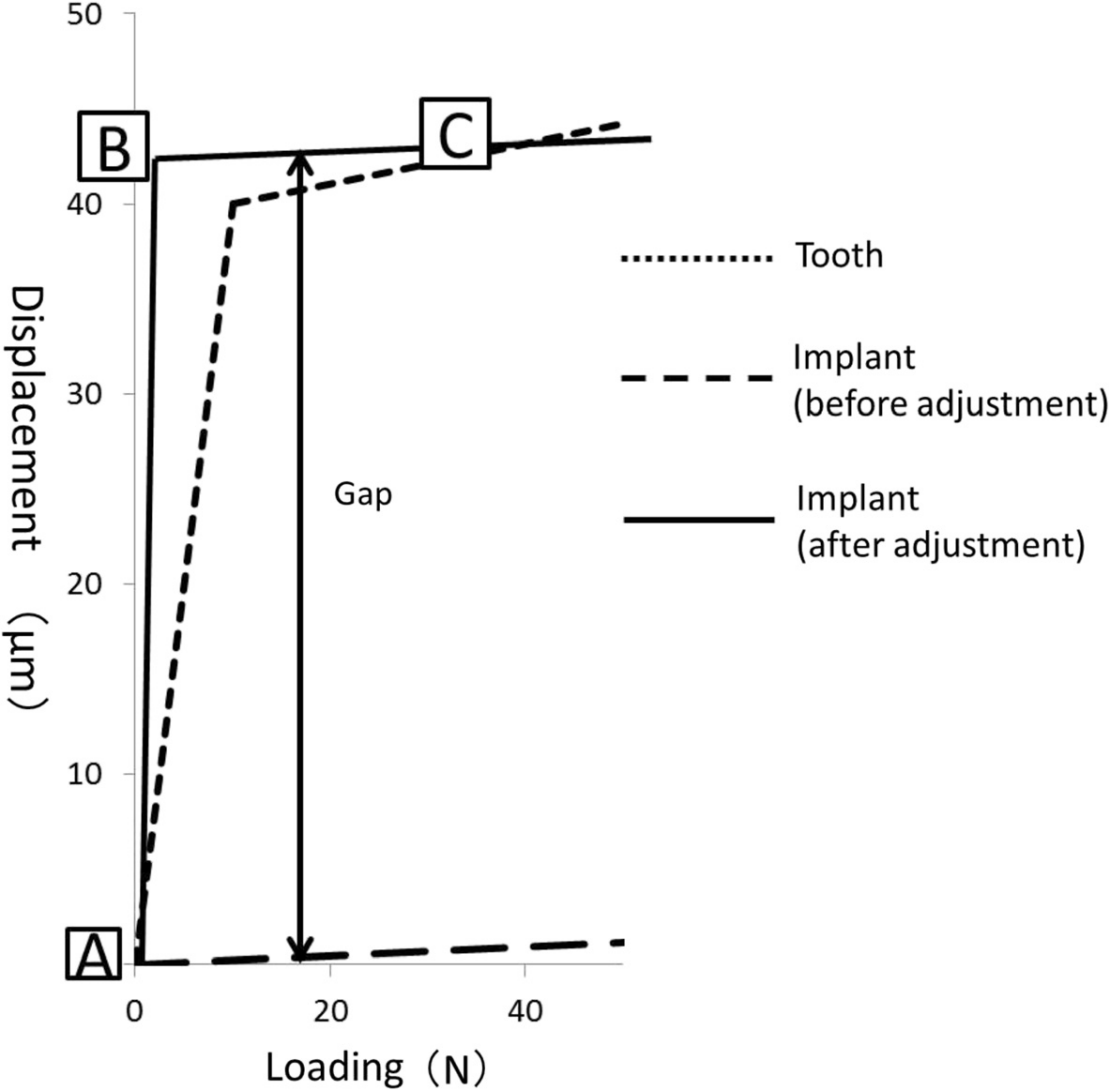
Occlusal adjustment was simulated by altering the load-displacement curves of the springs

**Fig. 5:**
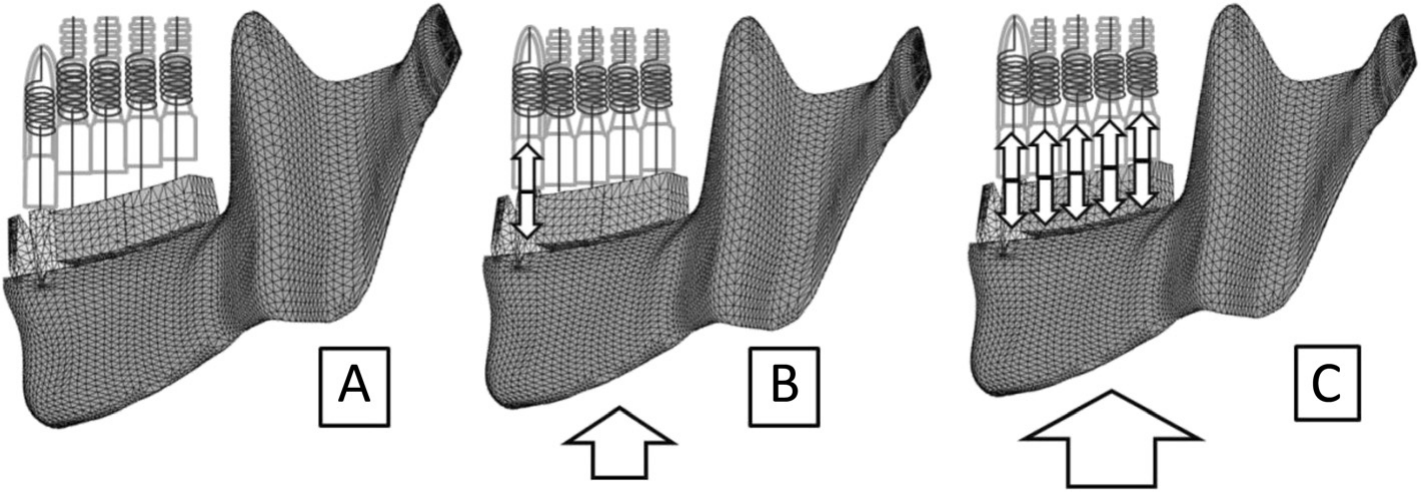
Schematic diagram for each phase of the load-displacement curve after occlusal adjustment of implants. **a**: Before loading, only anterior natural teeth were in contact with opposing teeth. Occlusal forces were not yet exerted anywhere. **b**: When a slight load caused the displacement of the mandible upward by the distance corresponding to the gap, i.e., the quantity of occlusal adjustment, the anterior teeth displaced into the socket and the implants were in contact with antagonists. Occlusal force was exerted only on anterior teeth. **c**: If the gaps were determined such that occlusal adjustment was completed, occlusal forces were distributed among natural teeth and implants under certain amounts of load

**Fig. 6:**
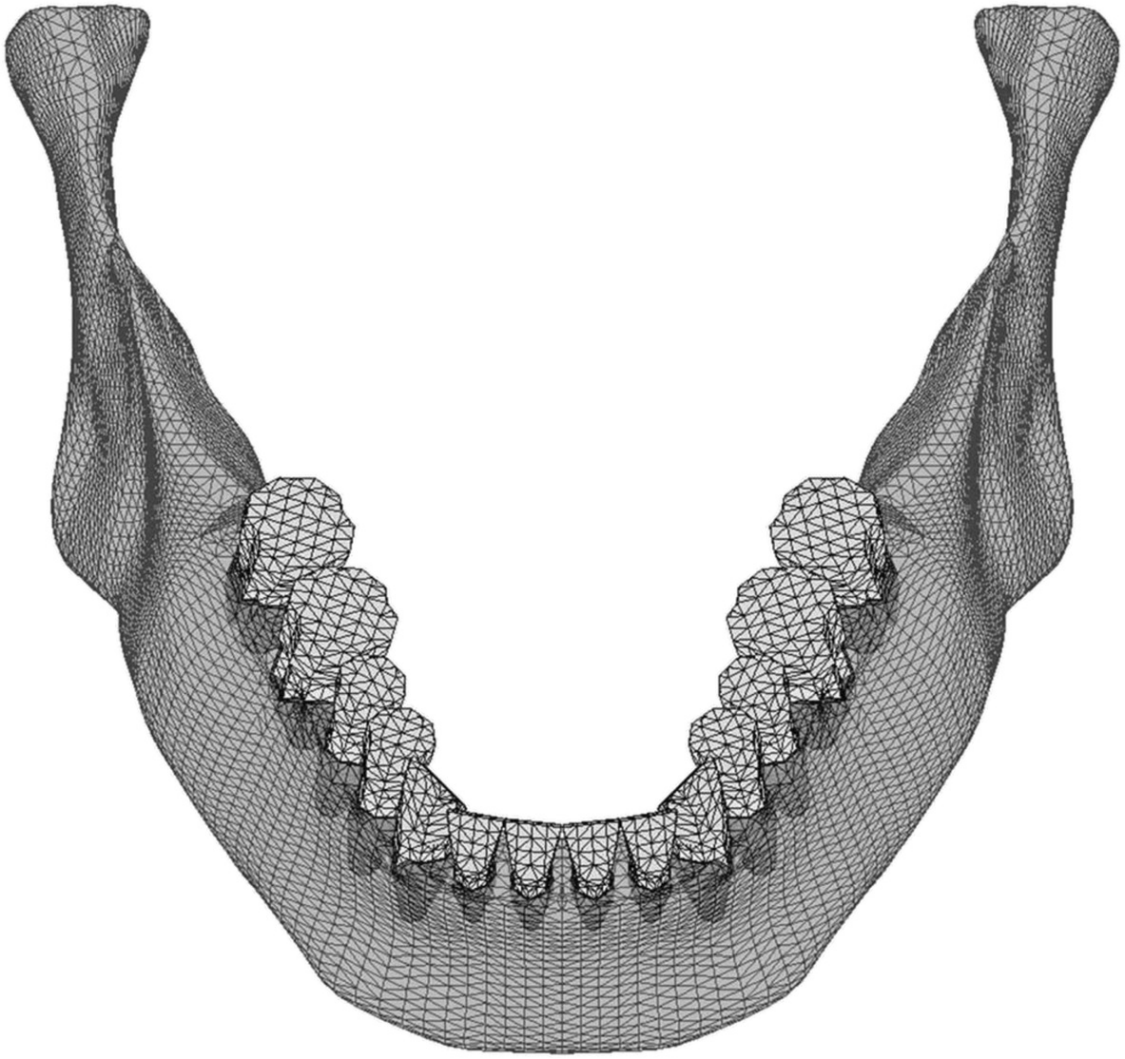
FE model with natural dentition (model-N). Tooth root is displayed with permeability

Occlusal adjustment was simulated by means of altering the load-displacement curves of the springs on the implants. The load-displacement curve was shifted so that the spring provided little resistance to compressive forces until the gap that was assumed to be made by occlusal adjustment closed (Figs. 3, 4, and 5). The size of each gap was decided by trial and error (Table 2) so that the occlusal force, i.e., the reaction force of the springs on the occlusal surface, was similar to that calculated with the FE model with natural dentition (model-N, Fig. 6).

**Table 2:** Size of each gap

Occlusal adjustment (model)	___	___	___	___
	4	5	6	7
Adj40N (model-T)	25.0	26.0	13.0	12.0
Adj200N (model-T)	30.0	37.0	23.5	24.0
Adj40N (model-I)	39.4	41.0	42.8	43.5
Adj200N (model-I)	70.9	75.4	79.9	81.6

### Loading conditions

The loading conditions assumed intercuspal clenching. On the assumption that occlusal force was generated by the contractile force of four bilateral masticatory muscles, the masseter, temporalis, mesial, and lateral pterygoid muscles, the loading points and the directions of the loads were determined based on the report by Korioth and Hannam [11] and anatomical findings [8–10]. The amount of the load was represented by the summation of the reaction forces at the occlusal surfaces of teeth in model-N. For example, the load condition that resulted in a total reaction force of 100 N in model-N was defined as Load100N.

### Procedure for analysis

The load conditions used during occlusal adjustment were Load40N, as a light bite (Adj40N), Load200N, as a hard bite (Adj200N), and Load400N, as the maximum biting force (Adj400N). Occlusal adjustment was performed through trial and error with reference to the distribution of the occlusal force calculated by FE analysis. When the similarities of the distribution of the reaction force on the superstructures to that on the natural teeth in model-N were confirmed, the occlusal adjustment was completed. Thereafter, the FE analysis was performed again under the load conditions of Load40N, Load100N, Load200N, Load400N, and Load800N using the FEA software package MSC.Marc2010 (MSC Software). The distributions of the reaction forces on the occlusal surface and on the mandibular condyle, which were regarded as the occlusal force and the load on the TMJ, respectively, were evaluated.

## Results

### Displaceability of teeth

**Fig. 7:**
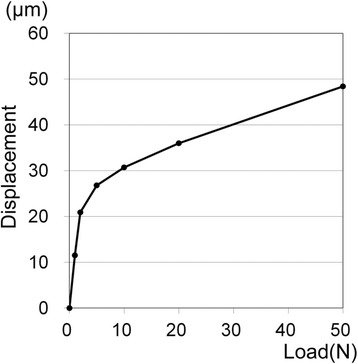
Load-displacement curve of the left canine

The load-displacement curve of the left canine under vertical load indicated two-phase displacement as shown in Fig. 7.

### Model-T

**Fig. 8:**
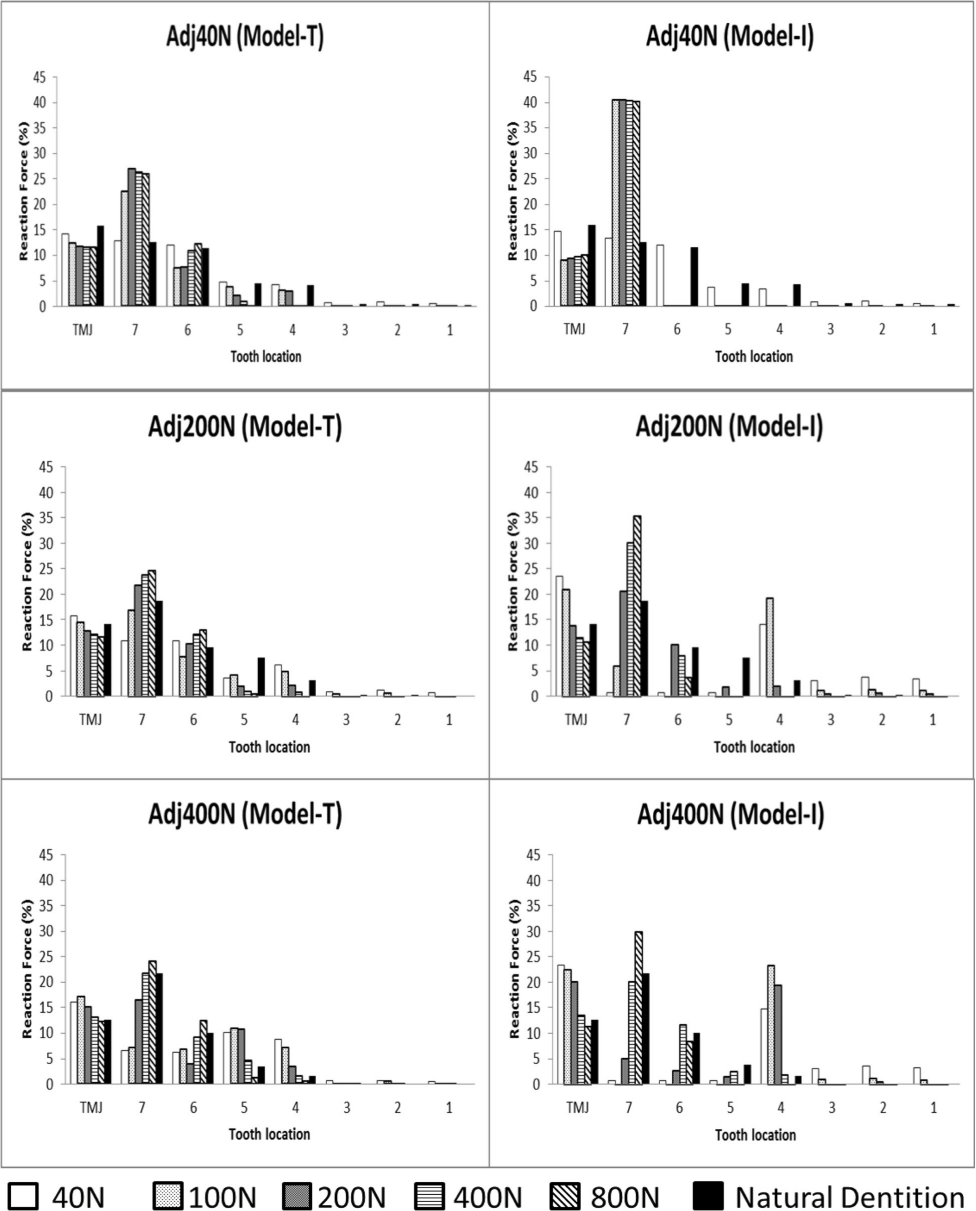
Distribution of the occlusal forces. *Left column*: model-T, *right column*: model-I, “Natural dentition” indicates the results in model-N under the load during occlusal adjustment

The results of model-T are shown in Fig. 8. Adj40N resulted in the concentration of approximately 25 % of the occlusal force at the most posteriorly located implant on each side. In other words, about half of the total occlusal force occurred at these implants under Load100N, Load200N, Load400N, and Load800N. At the premolar site implants, 6.9 and 4.8 % of the occlusal force was distributed under Load100N and Load200N, respectively. However, under Load400N and Load800N, occlusal force scarcely occurred there. The percentage of the total occlusal force (hereinafter abbreviated as POF) borne by the TMJ was smaller than that in model-N under all loading conditions.

Adj200N resulted in a smaller POF than in model-N at the implants in molar sites under Load40N and Load100N, which were conditions with less load than that exerted during occlusal adjustment. On the other hand, under these conditions, the occlusal force was larger than in model-N at the most anteriorly located implant. The POF in the TMJ was slightly larger than in model-N. Under Load400N, when the load was larger than that exerted during occlusal adjustment, 35.9 % of the occlusal force was concentrated at the molar site implants. Under Load800N, when the load was larger than that exerted during occlusal adjustment, 37.7 % of the occlusal force was concentrated at the molar site implants. In contrast, little or no occlusal force occurred at the premolar site implants. The POF in the TMJ was 12.1 % under Load400N and 11.7 % under Load800N.

Adj400N resulted in the reduction of POF at the molar site implants to half of the POF in model-N under Load40N and Load100N. On the other hand, 19.1 and 17.9 % of the occlusal force was distributed at the premolar site implants under Load40N and Load100N, respectively. The POF in the TMJ was 16.1 and 17.0 % under Load40N and Load100N, respectively. Under Load200N, 20.3 % of the occlusal force was distributed at the molar site implants and 14.0 % of the occlusal force was distributed at the premolar site implants. The POF in the TMJ was larger than that in model-N. Under Load800N, the POF at the molar site implants was 36.3 %. However, almost no occlusal force occurred at the premolar site implants and anterior teeth. The POF in the TMJ was almost the same as in model-N.

### Model-I

The results of model-I are shown in Fig. 8. Adj40N resulted in the concentration of approximately 40 % of the occlusal force at the most posteriorly located implant on each side under all loading conditions. In other words, about 80 % of the total occlusal force occurred at these implants. However, the occlusal force scarcely occurred at the premolar site implants and natural teeth. Around 10 % of the occlusal force was distributed at the TMJ. The POF was smaller than that in model-N.

Adj200N resulted in the concentration of the occlusal force at the most anterior implant under Load40N and Load100N. The POF in the anterior teeth and the TMJs was larger than that in model-N under Load40N and Load100N. Under Load400N, 38.0 % of the occlusal force was concentrated at the molar site implants. Under Load800N, 39.2 % of the occlusal force was concentrated at the molar site implants. Little occlusal force was present at the premolar site implants and natural teeth. At the TMJs, the POF was smaller than in model-N under Load400N and Load800N. Adj400N resulted in a concentration of occlusal force ten times larger at the most anterior implant than in model-N under Load40N, Load100N, and Load200N. The POF at the anterior teeth increased as the total occlusal load decreased. While the load was less than that exerted during occlusal adjustment, the POF at the most posterior implant was smaller than that in model-N. Under Load800N, 30.0 % of the occlusal force was concentrated at the most posterior implant. The POF in the TMJ was 23.3, 22.5, and 20.1 % under Load40N, Load100N, and Load200N, respectively.

## Discussion

### FE models

The FE models in this study were based on those reported by Kasai et al. [5]. The material properties of the soft tissues such as the PDL and the TMJ, which were mainly deformed in the analysis, were considered to be crucial, because the aim of this study was to investigate the distribution of occlusal forces on the teeth, implants, and TMJs. In Figs. 3 and 8, the PDLs of anterior teeth and the springs corresponding to opposing teeth show two-stage displaceability as reported previously [16, 17] and were considered to be appropriate. The load-displacement curve of the springs corresponding to TMJs was assumed to be similar to that of the cartilage [18] because of its far smaller elastic modulus than that of the TMJ disc [19, 20]. Therefore, the elastic modulus of the springs corresponding to TMJs was determined based on the thicknesses of the TMJ disc [21] and articular cartilage [19], the stress-strain curve of the intervertebral discs [18], and the displacement of the condyle [22, 23] in intercuspal clenching by indirect measurement. Although the material properties of human body depend on the individual, the models in this study were therefore considered to be appropriate to investigate the distribution of occlusal forces on the teeth, implants, and TMJs.

### The meaning of “occlusal adjustment” in this study

In the FE model before loading, there is no stress or deformation anywhere in the model with perfect even occlusal contact. However, this situation cannot really occur because of the existence of some occlusal load in the intercuspal position (ICP). Since the displaceability of dental implants is quite different from that of the natural teeth and TMJ, the distribution of the occlusal force exerted on the occlusal surface of natural teeth and superstructures depends on the amount of the occlusal load, i.e., the contractile force of the musculature. Thus, the “occlusal adjustment” performed on the FE models in this study was not a clinical procedure itself but a procedure to set the models in the state of the ICP under various occlusal loads. This problem can be clarified by the definition of the ICP itself. Although load and deformation of the bone, joints, periodontal ligaments, and teeth in the ICP depend on the amount of the occlusal load, its definition does not include how much occlusal load is appropriate to determine that a mandible is in the ICP [24]. The problem of occlusal adjustment of the superstructures on dental implants is, in a sense, deeply related to the definition of the ICP.

### Loading conditions

In this study, we selected the loading conditions assuming intercuspal clenching, because the effect of occlusal adjustment was considered to appear clearly. Based on the literature [25, 26], occlusal loading of 200 N was considered to correspond with a hard bite. The value for the “light bite” (40 N) was chosen so that the load intruded on all of the posterior teeth with a displacement corresponding to the midpoint of the first phase in the stress-displacement curve. This study was performed on the assumption that the maximum functional force was 400 N. Calculations were also performed under a load of 800 N, which was assumed to be the maximum nonfunctional occlusal force, such as that exerted in nocturnal bruxism. Because of the difficulty to control nocturnal bruxism, this value was considered to be sufficient to include the condition under the maximum force [27] as the load in bruxism.

### Effect of occlusal loading in occlusal adjustment and antagonists of implants

The occlusal force was concentrated on the most posterior implants while the load was larger under all loading conditions. This concentration of the occlusal force could be explained by the displaceability of TMJs. Since it was far larger than that of the teeth and implants (Fig. 3), the TMJs and ramus of the mandible were displaced upward and the most posterior implants became fulcrums of the rotation of the mandible. On the other hand, posterior implants were considered to be separated from opposing teeth and implants when the load was less than that exerted during occlusal adjustment. However, because of the smaller load itself, the actual occlusal force on the anterior implants was considered to be less harmful. The concentration of occlusal force was more marked in model-I than in model-T. This suggested the need for more careful occlusal adjustment in the case of opposing implants in both jaws because of the absence of the buffering effect of periodontal ligaments.

### Load bearing on TMJs

The percentage of bearing force at the TMJ was larger while the load was less than that exerted during occlusal adjustment, and vice versa. However, when the percentage of bearing force at the TMJ was large, the absolute force was not larger than in model-N under the load during occlusal adjustment, because the load itself was small. Therefore, the load borne by the TMJ was not considered to be harmful in any case of occlusal adjustment or load because the occlusal force itself was kept comparatively small even if the percentage of the bearing load increased.

### Suggestion of a clinical procedure for occlusal adjustment

In this study, when the load was larger than that exerted during occlusal adjustment, the concentration of the occlusal force in the molar region was considered to be harmful. However, since the occlusal force concentrated in the premolar region was relatively low when the load was less than that in occlusal adjustment, it was considered to be less harmful than in the former case. Therefore, according to our results, occlusal adjustment under maximum biting force was considered to be better to avoid the concentration of occlusal force on both implants and TMJs in Kennedy class I cases.

### Limitations of this study

It should be noted that these results were obtained under conditions of vertical loading by bilaterally balanced muscle activity with tight intercuspation in the correct mandibular position because the horizontal displacement of the premolars and molars was restrained. The actual distribution of occlusal forces may be different from the results of this study because there are individual differences in the material properties of the soft tissue. The lateral load, which may occur in lateral movement of the mandible during mastication, was not considered. These are problems that remain to be clarified in future research.

## Conclusions

Within the limitations of this study, it was concluded that the maximum biting force was better for occlusal adjustment with intercuspal clenching in bilateral distal extension of the superstructures on dental implants to prevent overloading of both TMJs and of the most posterior implants, especially in the case of opposing implants.
